# Comparison of corneal epitheliotrophic capacities among human platelet lysates and other blood derivatives

**DOI:** 10.1371/journal.pone.0171008

**Published:** 2017-02-02

**Authors:** Chien-Jung Huang, Yi-Chen Sun, Karen Christopher, Amy Shih-I Pai, Chia-Ju Lu, Fung-Rong Hu, Szu-Yuan Lin, Wei-Li Chen

**Affiliations:** 1 Department of Ophthalmology, National Taiwan University Hospital, Taipei, Taiwan; 2 Department of Ophthalmology, Taipei Tzu Chi General Hospital, The Buddhist Tzu Chi Medical Foundation, New Taipei City, Taiwan; 3 Department of Ophthalmology and Visual Sciences, Kellogg Eye Center, University of Michigan, Ann Arbor, Michigan, United States of America; 4 Sydney Medical School, University of Sydney, Sydney, Australia; 5 Center of Corneal Tissue Engineering and Stem Cell Biology, National Taiwan University Hospital, Taipei, Taiwan; 6 Department of Ophthalmology, Cathay General Hospital, Taipei, Taiwan; Cedars-Sinai Medical Center, UNITED STATES

## Abstract

**Purpose:**

To evaluate the corneal epitheliotropic abilities of two commercialized human platelet lysates (HPLs) and to compare the results with other blood derivatives, including human peripheral serum (HPS) and bovine fetal serum (FBS).

**Methods:**

*In vitro*, human corneal epithelial cells were incubated in various concentrations (0%, 3%, 5% and 10%) of blood derivatives. Two commercialized HPLs, including UltraGRO TM (Helios, Atlanta, GA) and PLTMax (Mill Creek, Rochester, MI), were tested and compared with HPS and FBS. Scratch-induced directional wounding assay was performed to evaluate cellular migration. MTS assay was used to evaluate cellular proliferation. Cellular differentiation was examined by scanning electron microscopy, inverted microscopy and transepithelial electrical resistance. Sprague-Dawley rats were used to evaluate the effects of the blood derivatives on corneal epithelial wound healing *in vivo*. Different blood derivatives were applied topically every 2 hours for 2 days after corneal epithelial debridement. The concentrations of epidermal growth factor (EGF), transforming growth factor -β1 (TGF-β1), fibronectin, platelet-derived growth factor-AB (PDGF-AB), PDGF-BB, and hyaluronic acid in different blood derivatives were evaluated by enzyme-linked immunosorbent assay (ELISA).

**Results:**

*In vitro* experiments demonstrated statistically comparable epitheliotropic characteristics in cellular proliferation, migration, and differentiation for the two commercialized HPLs compared to FBS and HPS. Cells cultured without any serum were used as control group. The epitheliotropic capacities were statistically higher in the two commercialized HPLs compared to the control group (p<0.05). Among the different concentrations of blood derivatives, the preparations with 3% yielded better outcomes compared to 5% and 10%. In rats, HPLs also caused improved but not statistically significant wound healing compared to HPS. All the blood derivatives had better wound healing ratios than the control group (p<0.05). In the quantification of epitheliotropic factors, UltraGRO and PLTMax had significantly higher levels of EGF, TGF- β1, fibronectin than human peripheral serum (p<0.05).

**Conclusions:**

Both commercialized HPLs showed comparable corneal epitheliotropic abilities and wound healing rates compared to HPS and FBS in the *in vivo* and *in vitro* studies. Our results suggest that HPLs may have the potential to replace HPS in the treatment of corneal epithelial problems.

## Introduction

Human peripheral serum (HPS) has long been used as a topical treatment for ocular surface disorders such as recurrent corneal erosions, persistent epithelial defects, superior limbic keratoconjunctivitis, and dry eye syndrome. [1–13] However, HPS has several major disadvantages. The process of obtaining peripheral blood from the patients and processing this to serum eye drops may be inconvenient for clinical application. The quality of HPS may be inconsistent, especially in patients with poor health, and the epitheliotropic abilities may be unsatisfactory or unpredictable. No standardized dilution protocol has been reported. HPS contains proinflammatory agents such as matrix metalloproteinase (MMP) and acid hydrolase that are derived from leukocyte degranulation and may induce unwanted side effects. [14, 15] In addition, HPS can be inconvenient for the patient due to its need to be stored at -4°C and be used preferably within a week. [16]

Human platelet lysate (HPL) is known to contain a number of mitogenic growth factors, including platelet-derived growth factor (PDGF), fibroblast growth factor (FGF), epidermal growth factor (EGF) and transforming growth factor (TGF) [17–19]. It is obtained from centrifugation and subsequent isolation of the platelet fraction from the platelet rich plasma (PRP). In order to induce platelet activation, the conversion of fibrinogen to fibrin clot, and growth factor release during the manufacturing process, platelet lysis is induced via either freeze-thaw cycles or the addition of CaCl_2_ [20] or thrombin[21–23]. Recently, it has been demonstrated that HPL can replace fetal bovine serum (FBS) and be used in mesenchymal stem cell (MSC) culture without adversely affecting the immunophenotype or MSC metabolism. [23–28] Since pharmaceutical grade HPLs are manufactured primarily from blood bank sources under strict Good Manufacturing Practices (GMP), the possibility of allogenic blood-derived infections is practically nonexistent. HPL can potentially replace HPS in treating ocular surface disorders, and be used as a substitute for FBS in MSC culture.

In this study, we hypothesized that HPL may provide comparable corneal epitheliotropic properties compared to HPS or FBS. We evaluated the rates of corneal epithelial proliferation, migration and differentiation *in vitro* with a human corneal epithelial cell line. The corneal epithelial wound healing abilities in an animal model were examined. We also compared the levels of several important corneal epitheliotropic factors in FBS, HPS, and HPL.

## Materials and methods

### Reagents and antibiotics

Dulbecco’s modified Eagle’s medium (DMEM), F12, Trypsin-EDTA, phosphate-buffered saline (PBS), FBS, and amphotericin B were purchased from Gibco (Rockville, MD). Dispase II was purchased from Roche Diagnostics Corporation (Indianapolis, IN). Enzyme-linked immunosorbent assay (ELISA) kit for TGF-β1 was purchased from RayBiotech, Inc. (Norcross, GA). ELISA kit for human EGF was obtained from eBioscience (San Diego, CA). Hyaluronic acid (HA) concentrations and PDGF-AB were measured using ELISA kits from R&D Systems (Minneapolis, MN). ELISA kit for fibronectin was purchased from Assaypro (Missouri, USA). ELISA kit for PDGF-BB was obtained from Peprotech (New Jersey, USA). All other reagents and chemicals were from Sigma-Aldrich (St. Louis, MO).

### Preparations of blood derivatives

#### Preparation of FBS

FBS was purchased from Gibco (Rockville, MD) and stored in sterile tubes at -20°C before use. It was diluted to 3, 5, and 10% in DMEM for *in vitro* cell culture experiments, and 3, 5, and 10% with Refresh Tear (Allergan, Inc. Parsippany, NJ) for topical use in animal experiments.

#### Preparation of HPS

The procedure used to obtain HPS was approved by the Institutional Review Board for Human Studies at the National Taiwan University Hospital (201510123RINB). All participants provided their written informed consent to participate in this study and the consent procedure was approved by the ethics committee. All individuals were healthy volunteers who were not taking any medication. Samples of 20 ml whole blood were drawn from 10 healthy volunteers (mean age, 30.3 ± 10.2 years) by venipuncture, allowed to clot at room temperature (20–25°C) for 4 hours, and centrifuged at 3000g for 15 minutes. The serum was then heated for 30 minutes at 56°C to eliminate further complement activation, carefully filtered and aliquoted in a sterile manner. The serum was stored in sterile tubes at -20°C before use. HPS was diluted to 3%, 5%, and 10% using similar methods as the FBS.

#### Preparation of HPL

Two commercialized HPLs, including UltraGRO TM (Helios, Atlanta, GA) and PLTMax (Mill Creek, Rochester, MI), were stored in sterile tubes at -20°C before use. The methods of HPL dilution to 3%, 5%, and 10% were similar to those of FBS.

### Culture of human corneal epithelial cell line (HCEC)

Human corneal epithelial cell line was purchased from ATCC (CRL-11515). The cells were centrifuged and then suspended in DMEM-F12 medium supplemented with antibiotic-antimycotic (100μg/ml penicillin/streptomycin and 1.25μg/ml amphotericin B) agents. Different percentages (3%, 5%, and 10%) of blood derivatives (HPS, HPLs, FBS) were added to the culture medium. The culture medium was replaced every 2 to 3 days. The cells were sub-cultured after reaching confluence. We used only cells from passages 2–3 of the initial culture. Cells cultured without any serum were used as control group.

### Cell-migration: Scratch-induced directional wounding assay

HCECs were plated in 12-well tissue culture dishes at a concentration of 4 x 10^5^ cells/ml and maintained in media with different concentrations (3%, 5%, and 10%) of different blood derivatives (HPLs, HPS, and FBS). After the cells reached confluence, a 200μl tip of the micropipette was used to wound the cells and create a linear scrape about 1 mm wide. Wound closure was recorded by photography at 0, 8, 12, and 16 hours after injury using an inverted microscope equipped with a digital camera (Diagnostic Instruments, Inc., Sterling Heights, MI). The wound closure was quantified with an image processing and analysis software program (Image J 1.37v; Wayne Rasband at the Research Services Branch, National Institute of Mental Health, Bethesda, MD) by measuring the average residual gaps between migrating cells of opposing wound edges. Wound healing ratio was defined as the difference between the initial and current cell-free areas divided by the initial cell-free area. All experiments were repeated six times to ensure consistent results.

### Cell proliferation: MTS assay

HCECs (5 x 10^3^/well) were loaded in 96-well plates and maintained in different concentrations (3%, 5%, and 10%) of blood derivatives (HPLs, HPS, FBS) for 3 days. After incubating for 24, 48, and 72 hours, the numbers of viable cells were determined with MTS assay (Promega corp., Madison, WI). According to the manufacturer’s instructions, the assay system measures the reduction of a 3-(4,5-dimethylthiazol-2-yl)-5-(3-carboxy-methoxyphenyl)-2-(4-sulfophenyl)-2H-tetrazolium inner salt (MTS) into a soluble formazan product by the mitochondria of viable cells. Since the production of formazan is proportional to the number of living cells, the intensity of the produced color is a good indicator of the cellular proliferative ability. Absorbances at 490 nm (test wavelength) and 650nm (reference wavelength) were measured using an ELISA microplate reader (Model ELx 800; Bio-TEK instruments. Inc., Winooski, VT). Wells containing culture medium but no cells served as controls. All experiments were repeated six times to ensure consistent results. [29]

### Cell differentiation-morphology: Inverted microscope and scanning electron microscopy (SEM)

4 x 10^5^ cells were seeded on 24-well plastic cell culture dishes with cover glasses and incubated for 3 days after confluency in media with different concentrations (3%, 5%, and 10%) of blood derivatives (HPLs, HPS, and FBS). After washing with PBS, specimens were fixed in 2.5% glutaraldehyde solution in 0.1M cacodylate buffer (pH 7.4) for 120 minutes, then post-fixed in 1% osmium tetroxide for 60 minutes and progressively dehydrated through ascending alcohol concentrations, critical point dried, mounted, and sputter coated with gold before examining with SEM (JSM6510LV; JEOL, Ltd, Tokyo, Japan). The surface morphology of the cells was evaluated by 2 independent examiners. All experiments were repeated 6 times.

### Cell differentiation-function: Measurement of transepithelial electrical resistance (TEER)

A total of 1 × 10^5^ cells incubated in 3% of different blood derivatives (HPLs, HPS, and FBS) were seeded in the upper chamber of a Costar transwell (Corning Costar, Cambridge, MA) (1.12 cm2 diameter, 0.4μm pore size) and allowed to reach confluency. TEER was measured using a Millicell-ERS electrical resistance system (Millipore, Bedford, MA) after the cells reach 80% confluency (day 0) and 3 days later (day 3) when the cells were at full confluency. The TEER values were calculated as Ω cm2 by multiplying it with the surface area (1.12cm2) of the monolayer. The resistance of the supporting membrane in the transwell filter is substracted from all readings before calculations. All experiments were repeated 6 times to ensure consistent results [29].

### Rat model of corneal epithelial wound healing

All animals in this study were handled according to the guidelines in the ARVO Statement for the Use of Animal in Ophthalmic and Vision Research. The protocol was approved by the Animal Care and Use Committee of National Taiwan University. All male Sprague-Dawley rats, 16–24 weeks, were purchased from Charles River Laboratories, Canada (n = 24). These animals were anesthetized with intraperitoneal injections of ketamine hydrochloride (2 mg/g body weight) and xylazine (0.4 mg/g body weight). The animals were monitored every 2 hour. After applying topical proparacaine (Alcaine; Alcon Laboratories, Inc., Fort Worth, TX) to each eye, the central cornea was marked by a trephine (4mm in diameter), and the epithelium was debrided by a corneal rust ring remover with a 0.5mm-burr (Algerbrush IITM; Alger Equipment Co., Inc., Lago Vista, TX) under the operating microscope (OPMI Pico I; Carl Zeiss Meditec, Jena, Germany). Blood derivatives (20% HPS, HPLs, FBS) were applied topically every 2 hours for 2 days. Rats treated with eye drops without blood derivatives were used as the control group. The area of corneal epithelial defect was checked at 0, 12, 24, and 48 hours with fluorescein staining and photographed under the operating microscope. Wound healing ratio was defined as the difference between the initial and current epithelial defect sizes divided by the initial epithelial defect area.

No animal died, appeared ill or suffered greatly prior to the experimental endpoints, although we had in place a protocol for early humane endpoints in cases where animals appeared irritable or in severe pain. Animals were maintained on a 12:12-hr light/dark cycle, and food and water were available *ad libitum* for the duration of experimentation.

### Quantification of epitheliotrophic factors

Quantification of epitheliotrophic factors was modified from the previously published method and performed in 3 different human blood derivatives[29] (2 HPLs and HPS). EGF, TGF-**β**1, fibronectin, PDGF-AB, PDGF-BB, and HA were measured by ELISA according to the manufacturer’s instructions.

### Chemical analysis

Contents of protein, glucose, chloride, sodium, potassium, calcium, phosphate, magnesium, iron, the total iron capacity, ferritin, vitamin B12, and folate in human peripheral serum and two commercialized human platelet lysates were determined using a Roche Module P800 Automatic Biochemical Analyzer (Roche Diagnstics, Mannheim, Germany).

### Data evaluation and statistical methods

ANOVA, the Dunnett’s multiple comparison test and the Student’s *t*-test were performed for statistical analysis. *P* value < 0.05 was considered statistically significant.

## Results

### Cell migration: Scratch-induced directional wounding assay

**Fig 1:**
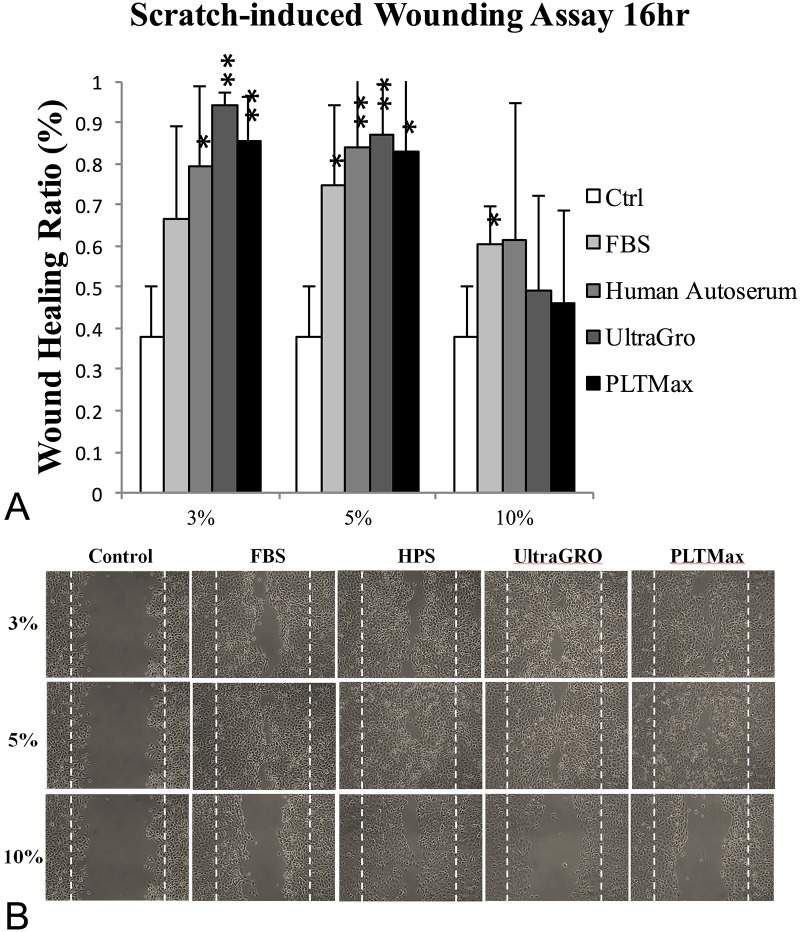
Cell migration: Scratch-induced directional wounding assay at 16 hours after wounding. (A) 10% preparations of blood derivatives demonstrated poorer response compared to 3% and 5% preparations. There was no significant difference among the 4 blood derivatives in 3% and 5% preparations. * indicated *p* <0.05. ** indicated *p* <0.01 compared to the control group that was without blood derivatives in culture media. (B) Representative picture of the effects of different blood derivatives on epithelial scratch wound healing at 16 hours after wounding. FBS: fetal bovine serum, HPS: human peripheral serum. UltraGRO: Human platelet lysate from Helios pharmaceutical. PLTMax: Human platelet lysate from Mill Creek pharmaceutical.

Fig 1 demonstrates the wound healing ratios at 16 hours. The healing ratios in 3% blood derivatives were 0.665±0.226, 0.791±0.195, 0.942±0.032, and 0.856±0.106 in FBS, HPS, UltraGRO, and PLTMax, respectively. The ratios at 16 hours in 5% blood derivatives were 0.745±0.197, 0.840±0.194, 0.868±0.122, and 0.827±0.203 in FBS, HPS, UltraGRO and PLTMax, respectively. In 3% and 5% blood derivative preparations, no significant differences were noted between HPS and the 2 commercialized HPLs. For the 10% blood derivatives, the wound healing ratios at 16 hours were 0.602±0.096, 0.612±0.336, 0.491±0.229, and 0.460±0.224 in FBS, HPS, UltraGRO, and PLTMax, respectively. A higher concentration (10%) appeared to retard wound healing for all 4 blood products. The inhibitory effect was more obvious in HPLs compared to that of HPS.

### Cell proliferation: MTS assay

**Fig 2:**
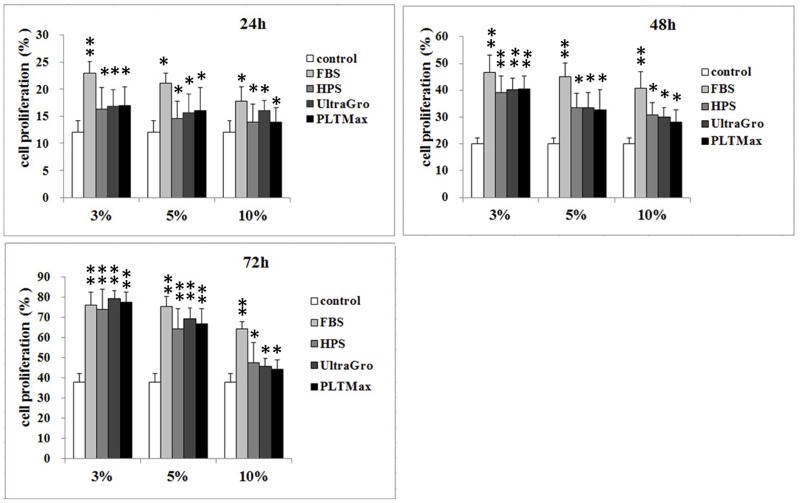
Cell proliferation: The effects of different blood derivatives on cellular proliferation with MTS assay. At 24 hours and 48 hours, corneal epithelial cells incubated with fetal bovine serum had significantly higher proliferative responses than those incubated in human peripheral serum, UltraGRO human platelet lysate (HPL), and PLTMax HPL. At 72 hours, there was no difference among these 4 products in 3% and 5% preparations. At 24, 48, and 72 hours, the proliferative responses showed no statistical difference between 3% and 5% blood derivative preparations. However, 10% preparation demonstrated poorer proliferative response compared to 3% and 5% preparations at all time points. FBS: fetal bovine serum, HPS: human peripheral serum. UltraGRO: Human platelet lysate from Helios pharmaceutical. PLTMax: Human platelet lysate from Mill Creek pharmaceutical. * indicated *p* <0.05. ** indicated *p* <0.01 compared to the control group that was without blood derivative in culture media.

Fig 2. demonstrates the results of the MTS assay. Similar to the cellular migration assay, there were no significant differences between HPS and the 2 HPLs in 3%, 5% and 10% preparations at all 3 time points. However, FBS seemed to have more cell proliferative ability compared to HPS and the 2 HPLs. In addition, higher concentration (10%) of HPS and the 2 HPLs showed more inhibitory effects on cell proliferation compared to that of FBS.

### Cell differentiation: Inverted microscopy, scanning electron microscopy (SEM) and transepithelial electric resistance (TEER)

**Fig 3:**
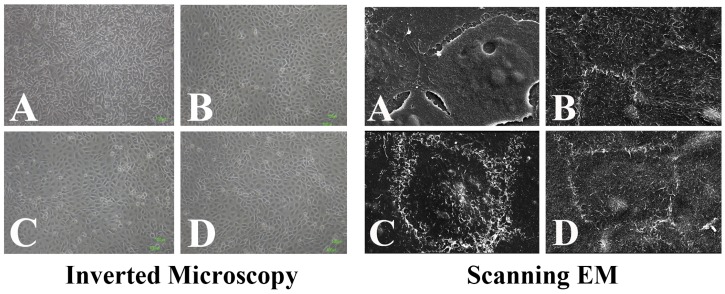
Cell differentiation: Inverted microscopy and scanning electron microscopy. Morphologies of human corneal epithelial cell (HCEC) lines cultivated for 48 hours with different blood derivatives, including (A) no serum, (B) human peripheral serum (HPS), (C) UltraGRO human platelet lysate (HPL), and (D) PLTMax HPL. Under inverted microscopy, cells incubated without serum formed a coherent monolayer of irregularly shaped cells (A) while cells cultivated in HPS, UltraGro, and PLTMax showed regular, polygonal flat cells (B-D). Under SEM, cells cultivated without serum showed increased cell-to-cell junctions without the formation of microvilli on the cell surface (A). The white arrow indicated the cell-to-cell junction and the white arrow heads represented the microvilli on the cell surface. Exfoliation of the cells from the culture dish was suspected in cells cultivated without blood derivatives. B-D) Cells cultivated in HPS, UltraGro, and PLTMax demonstrated tight cell-to-cell junction with upright microvilli homogenously and densely distributed at the cellular surface. Original magnifications of the inverted microscopy: 100X and SEM 1250X. UltraGRO: Human platelet lysate from Helios pharmaceutical. PLTMax: Human platelet lysate from Mill Creek pharmaecutical.

**Fig 4:**
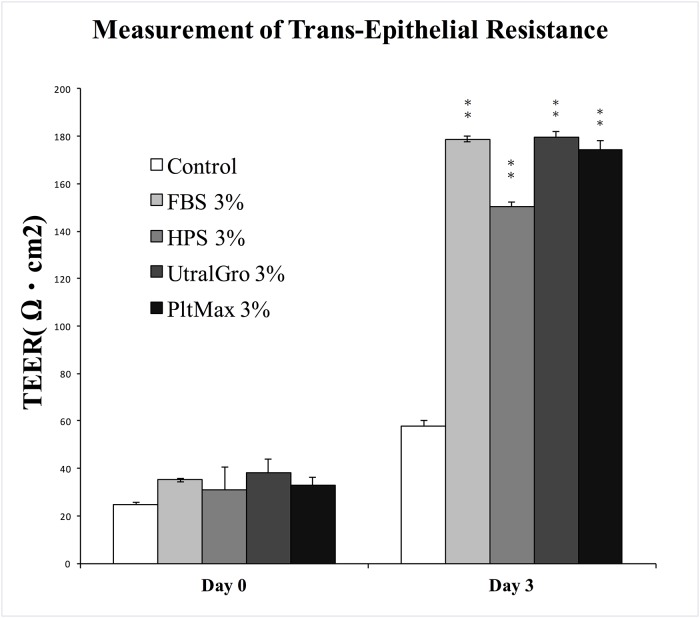
Cell differentiation: Measurement of trans-epithelial electrical resistance (TEER). The effects of different blood derivatives on trans-epithelial electrical resistance. Cells incubated with fetal bovine serum (FBS), human peripheral serum (HPS), UltraGRO human platelet lysate (HPL), and PLTMax HPL demonstrated significantly higher TEER values compared to the control group. However, there was no significant differences among FBS, HPS, UltraGRO, and PLTMax on day 3 after the cells reached confluency. UltraGRO: Human platelet lysate from Helios pharmaceutical. PLTMax: Human platelet lysate from Mill Creek pharmaceutical. * indicated p <0.01. ** indicated p <0.01 compared to the control group that was without any blood derivative.

After incubating HCECs with 3% of various blood preparations for 3 days, cellular differentiation patterns were evaluated by inverted microscopy, SEM (Fig 3) and TEER (Fig 4). Under the inverted microscope, cells incubated without serum (Fig 3A) showed a coherent monolayer of irregularly shaped cells that were significantly different from normal cultivated epithelial cell morphology. In contrast, cells incubated with HPS and HPLs showed a coherent monolayer of cells with the regular polygonal morphology quite similar to that of normal cultivated epithelial cells (Fig 3B–3D). Under SEM, cells cultivated without serum showed increased cell-to-cell junctions without the formation of prominent microvilli on the cellular surface. Partial exfoliation of the cell borders from the culture dish was suspected (Fig 3A). Cells cultivated in HPS and the 2 HPLs demonstrated tight cell-to-cell junctions with prominent upright microvilli homogenously and densely distributed at the cellular surface (Fig 3B–3D). TEER is a functional differentiation assay which can reflect the epithelial tightness and functional integrity. In our study, cells cultivated in 3% of blood derivatives showed no significant difference in TEER values among HPS and the 2 HPLs on day 0 and day 3 of initial measurements. [30] (Fig 4)

### Rat model of corneal epithelial wound healing

**Fig 5:**
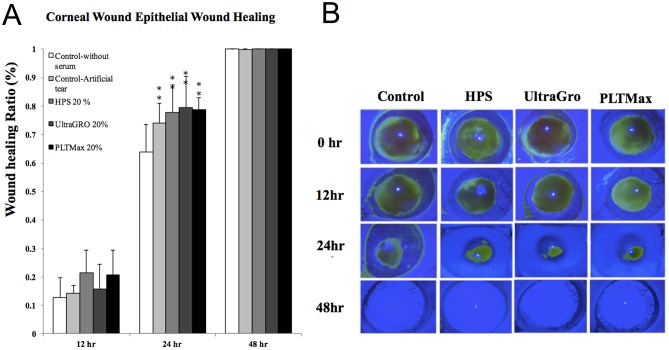
Rat model of corneal epithelial wound healing. (A) *In vivo* rat corneal epithelial wound healing after epithelial debridement and topical treatment with 20% of human peripheral serum (HPS) and the 2 different human platelet lysates (HPLs). There were significantly better wound healing abilities in HPS and the 2 HPL groups compared to the control group at 24 hours after wounding. (B) Representative pictures of corneal epithelial defects at 12, 24 and 48 hours after surgery in rat eyes that received epithelial debridement and different treatments. UltraGRO: Human platelet lysate from Helios pharmaceutical. PLTMax: Human platelet lysate from Mill Creek pharmaceutical. * indicated *p*<0.05 which was compared to the control group without any blood derivative.

Fig 5 demonstrated the *in vivo* rat corneal epithelial wound healing after epithelial debridement and topical treatment with 20% of HPS and the 2 HPLs. The wound healing ratios at 24 hours were 0.639±0.095, 0.740±0.069, 0.778±0.096, 0.794±0.110, and 0.786±0.043 in control, FBS, HPS, UltraGRO, and PLTMax, respectively. There were significant increases in corneal epithelial wound healing in both the HPS and the 2 HPL groups compared to the control group 24 hours after injury (*p*<0.05).

### Quantification of epitheliotropic factors and chemical analysis

Quantification of epitheliotropic factors was performed in HPS and the 2 HPLs. Table 1 illustrates the ELISA assay results of EGF, TGF-**β**1, PDGF-AB, PDGF-BB, HA, and fibronectin. The concentrations of EGF, TGF-**β**1, PDGF-AB, PDGF-BB, and HA were higher in the HPL groups compared to those in HPS, but fibronectin was significantly lower in the HPL groups. Chemical analysis (Table 2) revealed different concentrations of chemicals among the different groups, with significantly more glucose and significantly less ferritin in the PLTmax compared to HPS.(p<0.05)

**Table 1:** Concentrations of epitheliotropic factors in different human blood preparations (n = 3).

Blood Preparations	HPS	UltraGRO	PLTMax
**EGF (ng/ml)**	0.67±0.01*^§^	5.89±0.09*^†^	7.53±0.42^§^^†^
**TGF- β1 (ng/ml)**	57.79±0.85*^§^	98.35±0.26*^†^	107.07±0.09^§^^†^
**Fibronectin (μg/ml)**	566.54±63.31*^§^	76.60±20.03*	147.06±3.71^§^
**PDGF-AB (ng/ml)**	0.80±0.05*^§^	1.47±0.01*	1.72±0.01^§^
**PDGF-BB (ng/ml)**	0.87±0.38^§^	3.53±2.83	10.54±0.68^§^
**Hyaluronic Acid(ng/ml)**	32.23±0.22^§^	42.04±3.33^†^	66.29±4.11^§^^†^

*Significant difference between HPS and UltraGRO(p<0.05)

^§^ Significant difference between HPS and PLTMax (p<0.05)

^†^Significant difference between UltraGRO and PLTMax (p<0.05)

UltraGRO: Human platelet lysate from Helios pharmaceutical. PLTMax: Human platelet lysate from Mill Creek pharmaceutical.

**Table 2:** Comparative chemical analysis of fetal bovine serum (FBS), human peripheral serum (HPS), and 2 commercialized human platelet lysates (UltraGRO, PLTMax) (n = 3).

	FBS	HPS	UltraGRO	PLTMax
Glucose(mg/dI)	101.7±30.0	82.3±13.1^§^	129.0±33.1	200.0±3.6^§^
Chloride(mEq/1)	99.3±0.6	104.3±1.2*^§^	115.7±2.5*^†^	83.3±0.6^§^^†^
Sodium(mEq/1)	136.3±1.2	141.7±2.5*^§^	155.3±3.21*	160.0±1.0^§^
Potassium(mEq/l)	>10.0	4.3±0.2*^§^	5.0±0.1*	4.8±0.1^§^
Calcium(mg/dI)	14.0±0.1	9.0±0.3*	43.1±5.3*^†^	7.8±0.3^†^
Phosphate(mg/dI)	10.5±1.0	3.4±0.3	4.7±0.21	4.6±0.1
Magnesium(mg/dI)	3.4±0.1	1.9±0.3	2.2±0.1	2.0±0.0
Iron(ug/dI)	207.3±40.5	122.7±18.2	69.7±10.2	73.7±4.9
Ferritin(ng/ml)	0.8±0.2	70.2±24.4^§^	37.2±6.5	25.3±2.6^§^
Vitamin B12(pg/ml)	205.7±14.3	523.0±125.4	505.7±53.0	437.0±83.6
Folate(ng/ml)	8.5±6.5	5.8±1.6	3.9±2.3	7.5±1.9

*Significant difference between HPS and UltraGRO (p<0.05)

^§^ Significant difference between HPS and PLTMax (p<0.05)

^†^Significant difference between UltraGRO and PLTMax (p<0.05)

TIBC: total iron-binding capacity; UIBC: unsaturated iron-binding capacity

UltraGRO: human platelet lysate from Helios pharmaceutical. PLTMax: human platelet lysate from Mill Creek pharmaceutical

## Discussion

In this study, we demonstrated two different commercialized HPLs with corneal epitheliotropic capacities not inferior to that of HPS both in vivo and in vitro. Commercialized HPLs had significantly higher concentrations of several important growth factors, such as EGF, TGF-β1, PDGF-AB and PDGF-BB, compared to HPS. As far as we know, this is the first study to evaluate commercialized HPLs and to compare their corneal epitheliotropic properties with those of other blood derivatives. We believed these two HPLs have the potential to be used as topical eye drops for facilitating corneal re-epithelialization and to replace blood derivatives like HPS.

Corneal wound healing is a complex process, which involves cellular migration, proliferation, differentiation, and deposition of extracellular substances.[29] In this study, we investigated the corneal epithelial migration, proliferation, and differentiation in 4 different blood-derived preparations, including two commercialized HPLs (UltraGRO and PLTMax), HPS, and FBS, in an *in vitro* cell culture model and in an *in vivo* rat corneal epithelial wound healing model. FBS, a well-known and widely used calf derived blood product, was used as control in our study. During the process of corneal epithelial wound healing, the initial migration of epithelial cells that cover the denuded area occurs before cellular proliferation and differentiation[31]. Scratch-induced directional wounding assay was designed to detect the effects of blood derivatives on cellular migration *in vitro*. HPS and the 2 HPLs had better capabilities to promote cellular migration than FBS (in 3% and 5% preparation), and the 2 HPLs were not inferior to HPS. Interestingly, a higher concentration (10%) produced significantly poorer cellular migration in all blood preparations compared to lower concentrations, especially in the 2 commercialized HPLs. These results seemed to imply that lower concentrations (3% and 5%) of HPLs promote better corneal epithelial cell migration, while higher concentrations may worsen cellular migration as shown *in vitro*. (Fig 1) This finding is of interest for clinical application since it may decrease the demands for a higher concentration of HPL supplies. We also performed the MTS assay to evaluate the cellular proliferating ability among different blood derivatives and found that the result was similar to that of the scratch-induced directional wound healing assay. There was no significant difference in cellular proliferating abilities between HPS and the 2 HPLs. Higher concentration (10%) once again resulted in poorer cellular proliferation. The reason why higher concentrations of HPS and HPLs cause poorer corneal epithelial cell migration and proliferation may be due to TGF-β, an anti-proliferative cytokine which can inhibit cell proliferation in a dose-dependent manner. [32] We found that HPLs contained higher concentrations of TGF-β than HPS, which may explain the higher inhibitory effect found in 10% HPLs than in 10% HPS. Some reports have shown satisfactory cornea epitheliotropic effects of 50% and 100% HPS in treating dry eye diseases. [33–35] Our study revealed that lower concentrations of HPS (3% and 5%) had better epitheliotropic abilities than a higher concentration (10%) *in vitro*. Further *in vivo* studies may be required to confirm this finding.

We also performed inverted microscopic imaging, SEM, and TEER to identify cellular differentiation in 3% HPS and HPLs. Under inverted microscopy and SEM, the cells cultured without any blood derivative did not differentiate well, while the cells cultured in the three human blood derivatives (HPS and 2 HPLs) had regular polygonal shapes, compact cell-to-cell junctions, and prominent upright microvilli homogenously and densely distributed at the cellular surface, showing signs of good differentiation. The TEER result of the cells cultivated with 3% blood derivatives also showed similar results as SEM. We demonstrated consistent and reliable results via cellular morphologies detectable by SEM, and functions of intercellular junctions as measured by TEER, reinforcing that 3% HPLs may be able to replace HPS.

In addition to the *in vitro* cell culture system, which investigated cellular migration, proliferation, and differentiation separately, we performed an *in vivo* corneal epithelial wound healing assay using Sprague-Dawley rats to reflect the physiological healing process. The *in vivo* results supported the *in vitro* results, showing that HPS and HPLs had comparable effects in promoting corneal epithelial wound healing.

The reason for using blood-derived products (e.g. HPS and HPL) as topical eye drops in corneal epithelial disorders is mainly due to the existence of abundant growth factors. [36–39]. Growth factors can be released by platelet activation and this can be reproduced in vitro to prepare growth factor-rich fluids like PRP. We compared several important epitheliotropic factors among HPS and the 2 commercialized HPLs. Our results showed that the 2 HPLs contained significantly higher concentrations of EGF, TGF-**β**1, PDGF-AB, PDGF-BB, and HA compared to HPS. EGF is normally secreted by lacrimal glands and corneal epithelial cells, and is well known to exert potent proliferative effects on the corneal epithelium [40, 41]. TGF-**β**1 can inhibit corneal epithelial cell proliferation but has been suggested to play an important role in cellular differentiation and migration [29, 42]. TGF-**β**1 also stimulates corneal epithelial cell migration via the activation of integrin-**β**1.[43, 44] Fibronectin is a glycoprotein that supports cell adhesion and is an important mediator for cellular migration. With the presence of fibronectin, PDGF isoforms can stimulate migration of rabbit corneal epithelial cells. [45] All the factors mentioned above are important for corneal wound healing. In this study, various concentrations of these epitheliotropic components were measured in the HPS and the 2 HPLs. We did not evaluate the concentrations in FBS due to the incomparability secondary to species differences. However, wound healing is a complex process and it can be difficult to tell which single epitheliotropic factor might predominantly control wound healing [29].

Topical application of HPS is commonly used in patients with poor epithelial healing. However, autologous HPS has laborious processing time and other drawbacks that limit its clinical use. [29]. Furthermore, the presence of autoantibodies in several ocular autoimmune diseases, such as systemic lupus erythematosus, Sjogren’s syndrome, and graft-versus-host disease, can decrease efficacy in treating ocular surface diseases. [46] In 2011, Shen EP et al. reported that human cord blood serum was superior to HPS in promoting corneal epithelial proliferation and differentiation. [29] However, the difficulty in obtaining human cord blood serum limits its applicability.

Platelets are specialized cells with biologically active substances such as various growth factors that are released from intracellular alpha granules when activated. They play an important role in the process of wound healing. [47, 48] The most well-known platelet-derived growth factors include PDGF, TGF-α, TGF-β, FGF, and vascular endothelial growth factors (VEGF). Although some of these growth factors are available in purified forms, wound healing is a complex process and cannot be mediated by a single agent. Growth factors obtained from platelets may play a role in regulating epithelial healing. [48, 49]. Recently, HPL has been proposed as an alternative for the treatment of various diseases and as a replacement for FBS during *ex vivo* stem cell expansion.[50] Human blood products are devoid of immunogenic risks due to their human origin. Secondly, the infrastructure for blood collection, as well as the quality and safety tests, are well-established in most developed countries. In countries like the United States, Germany, Switzerland, France, blood products are regulated as pharmaceutical/medicinal products and manufactured under the principle of GMP, which contributes to optimized product consistency, viral safety, and traceability. Since the World Health Organization (WHO) guidelines encourage the GMP implementation in blood establishments at a global level, this should increase the availability of qualified sources [50, 51].

Although autologous HPLs have been commonly reported in the treatment of corneal epithelial disorders, the use of commercialized HPLs can provide additional benefits. Firstly, a large amount of supply can be provided to many patients. Growth factors obtained from platelets may play a role in regulating epithelial healing. Secondly, conventional human peripheral serum needs to undergo 2 hours of precipitation for clotting, centrifugation for 15 minutes, and subsequent dilution of serum to 20 percent with BSS. The whole process may take hours. Commercialized HPL-derived eye drops can be prepared in advance and do not require blood draws or processing of the serum, thus shortening the patient waiting time. Finally, patients with Stevens-Johnson syndrome, bullous pemphigoid or severe graft-versus-host-disease often need long term use of human peripheral serum eye drops and can suffer from repeated blood draws for human peripheral serum. Commercialized HPLs will not require blood draws in these patients.

To our knowledge, this is the first study to systematically compare corneal epitheliotropic effects of commercialized HPLs with that of HPS. There are some limitations in our study. Firstly, the *in vitro* culture system itself is still a different environment compared to the *in vivo* system. The complex physiological and molecular interactions of the tear film and ocular surface *in vivo* cannot be completely replicated by cell culture models. Besides, the human corneal epithelial cell line used in this study may be tumorgenic and different from normal corneal epithelium since it was immortalized with SV-40 virus transformation. Further in vitro experiments using primary cultivated corneal epithelial cells or in vivo experiments are needed to support the current study. [52] Secondly, the concentrations and frequencies of autologous serum eye drops application may affect outcomes and these were different for *in vitro* models, which had continuous exposure to serum. Finally, we used denuded rat cornea as an *in vivo* model. However, the wound healing process of healthy rat corneas can be different from the diseased human corneas.

In conclusion, we found that commercialized HPLs can promote corneal epithelial wound healing in both *in vivo* and *in vitro* experimental systems. Commercialized HPLs may have the potential to replace HPS eye drops in the treatment of various ocular surface disorders.
